# Supplementary figures and images for: Effects of systemic oxytocin and beta‐3 receptor agonist (CL 316243) treatment on body weight and adiposity in male diet‐induced obese rats
Source: Front Endocrinol (Lausanne). 2025 Mar 4;16:1503096. doi: 10.3389/fendo.2025.1503096 (PMC11913664; doi:10.3389/fendo.2025.1503096)

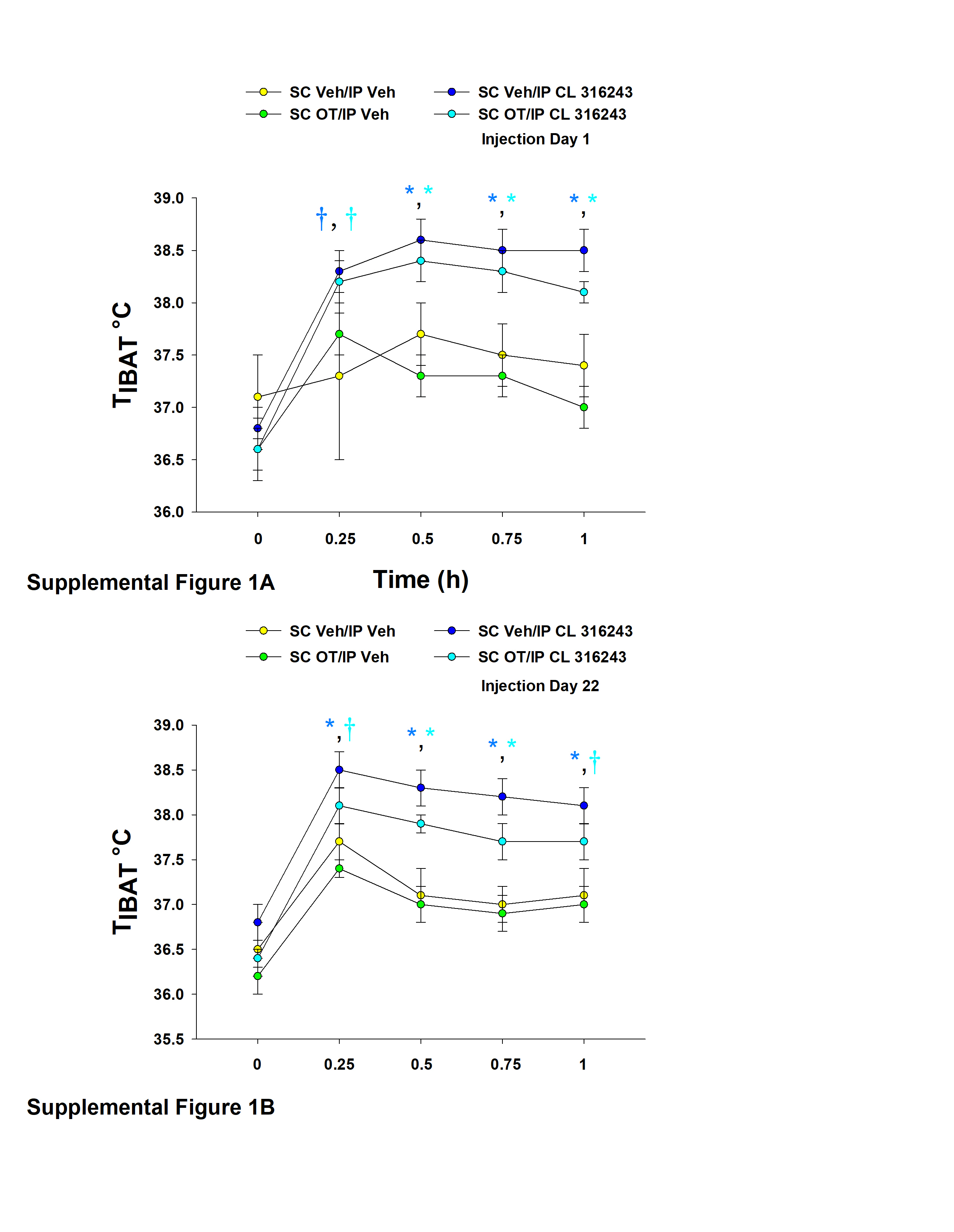

Supplement: Supplementary Figure 1 — (A, B) TIBAT measurements following acute systemic administration of the beta‐3 receptor agonist (CL 316243) or vehicle in male DIO rats. (A), injection day 1 (0.5 mg/kg) and (B), injection day 22 (0.5 mg/kg). Data are expressed as mean ± SEM. *P<0.05 vs VEH; †0.05<P<0.1 vs VEH. [file Image1.jpg]

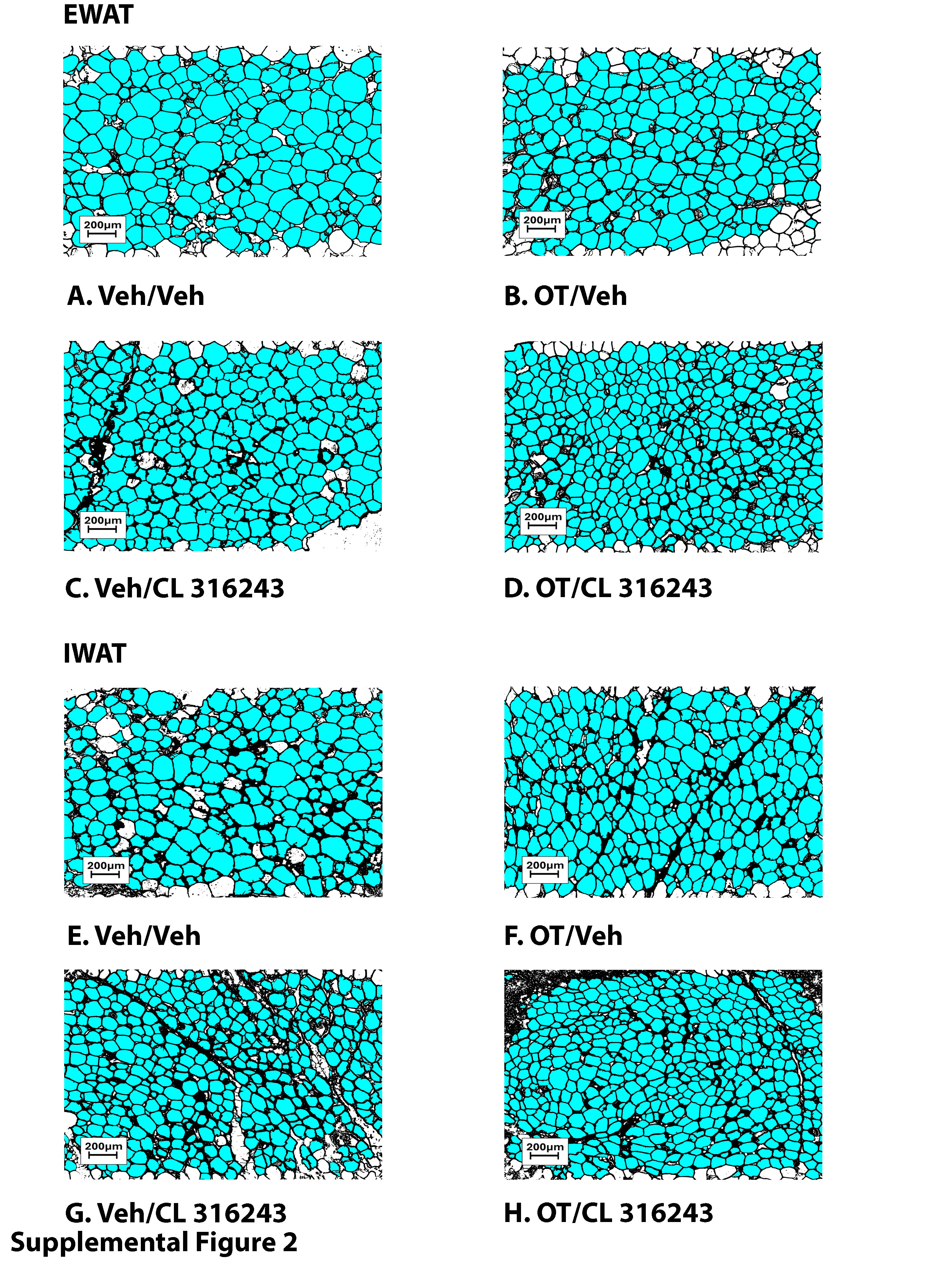

Supplement: Supplementary Figure 2 — (A–H): Representative image to illustrate the effect of chronic systemic OT infusions (50 nmol/day) and systemic beta-3 receptor agonist (CL 316243) administration (0.5 mg/kg) on adipocyte size in EWAT and IWAT in male DIO rats. Adipocyte size was analyzed using ImageJ. Images were taken from fixed (4% PFA) paraffin embedded sections (5 μm) containing EWAT (A–D) or IWAT (E–H) in HFD-fed rats treated with systemic OT (50 nmol/day) or vehicle in combination with IP CL 316243 (0.5 mg/kg) or IP vehicle. A/E, Veh/Veh. B/F, OT/Veh. C/G, Veh/CL 316243. D/H, OT-CL 316243; (A–H) all visualized at 100X magnification. [file Image2.jpg]

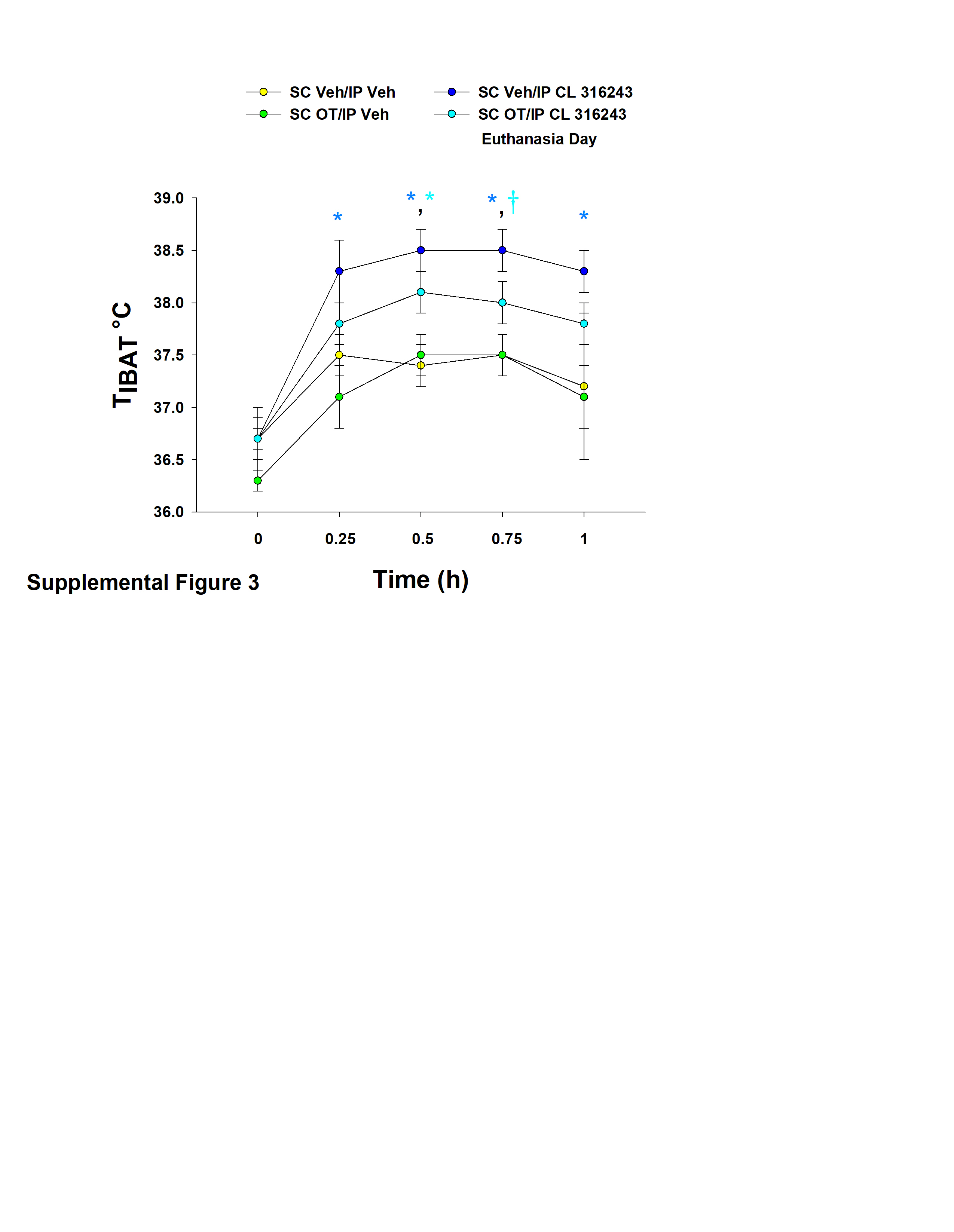

Supplement: Supplementary Figure 3 — TIBAT measurements following acute systemic administration of the beta‐3 receptor agonist (CL 316243) or vehicle in male DIO rats. A, injection day 23 prior to euthanasia (0.5 mg/kg), euthanasia day. Data are expressed as mean ± SEM. *P<0.05 vs VEH; †0.05<P<0.1 vs VEH. [file Image3.jpg]
